# An epidemic CC1-MRSA-IV clone yields false-negative test results in molecular MRSA identification assays: a note of caution, Austria, Germany, Ireland, 2020

**DOI:** 10.2807/1560-7917.ES.2020.25.25.2000929

**Published:** 2020-06-25

**Authors:** Stefan Monecke, Elisabeth König, Megan R Earls, Eva Leitner, Elke Müller, Gabriel E Wagner, David M Poitz, Lutz Jatzwauk, Teodora Vremerǎ, Olivia S Dorneanu, Alexandra Simbeck, Andreas Ambrosch, Ines Zollner-Schwetz, Robert Krause, Werner Ruppitsch, Wulf Schneider-Brachert, David C Coleman, Ivo Steinmetz, Ralf Ehricht

**Affiliations:** 1 Leibniz Institute of Photonic Technology (IPHT), Jena, Germany; 2 Institute for Medical Microbiology and Hygiene, Medical Faculty ‘Carl Gustav Carus’, Technische Universität Dresden, Dresden, Germany; 3 InfectoGnostics Research Campus Jena, Jena, Germany; 4 These authors contributed equally; 5 Diagnostic and Research Institute for Hygiene, Microbiology and Environmental Medicine, Medical University of Graz, Graz, Austria; 6 Microbiology Research Unit, Division of Oral Biosciences, Dublin Dental University Hospital, Trinity College, University of Dublin, Dublin, Ireland; 7 Institute of Clinical Chemistry and Laboratory Medicine, University Hospital ‘Carl Gustav Carus’, Technische Universität Dresden, Dresden, Germany; 8 Department of Hospital Infection Control, University Hospital ‘Carl Gustav Carus’, Technische Universität Dresden, Dresden, Germany; 9 Microbiology Unit, Department of Preventive and Interdisciplinary Medicine, University of Medicine and Pharmacy ‘Grigore T Popa’, Iaşi, Romania; 10 Department of Infection Control and Infectious Diseases, University Hospital Regensburg, Regensburg, Germany; 11 Institute of Laboratory Medicine, Microbiology and Hygiene, Barmherzige Brüder Hospital, Regensburg, Germany; 12 Section of Infectious Diseases and Tropical Medicine, Department of Internal Medicine, Medical University of Graz, Austria; 13 Institute of Medical Microbiology and Hygiene, Austrian Agency for Health and Food Safety, Vienna, Austria; 14 Friedrich- Schiller University; Institute of Physical Chemistry, Jena, Germany

**Keywords:** Staphylococci, Staphylococcus aureus, CC1-MRSA-IV, commercial PCR, Cepheid GenXpert, BD MAX, next generation sequencing

## Abstract

We investigated why a clinical meticillin-resistant *Staphylococcus aureus* (MRSA) isolate yielded false-negative results with some commercial PCR tests for MRSA detection. We found that an epidemic European CC1-MRSA-IV clone generally exhibits this behaviour. The failure of the assays was attributable to a large insertion in the *orfX*/SCC*mec* integration site. To ensure the reliability of molecular MRSA tests, it is vital to monitor emergence of new SCC*mec* types and junction sites.

We investigated why a clinical meticillin-resistant *Staphylococcus aureus* (MRSA) isolate, collected in Austria in late 2019, yielded false-negative results with two widely used commercial *orfX*/SCC*mec* junction assays (Cepheid GeneXpert MRSA/SA BC, BD MAX Staph SR). The aim of this study was to investigate false-negative results with these two assays. Therefore, we tested and sequenced the index isolate and other isolates assigned by microarray to the same strain (i.e. the European CC1-MRSA-IV [1]).

## Index case

A 62-year-old patient with metastasised cancer was admitted with suspected pneumonia to the Medical University Hospital of Graz, Austria. Blood cultures (BACTEC, Becton Dickinson, Heidelberg, Germany) became rapidly positive and *Staphylococcus aureus* was identified by in situ hybridisation (PNA FISH, AdvanDx, Woburn, United States (US)). In order to identify meticillin-resistant *S. aureus* (MRSA), the blood culture was investigated using the GeneXpert MRSA/SA BC PCR (Cepheid, Sunnyvale, US). Simultaneously, rapid antimicrobial susceptibility testing (RAST) was performed [2,3]. The GeneXpert test was negative for MRSA but RAST revealed cefoxitin resistance after 6 h. Antimicrobial susceptibility testing (Vitek2, bioMérieux, Marcy-l’Étoile, France) confirmed meticillin resistance. BD MAX StaphSR (Becton Dickinson) yielded a negative MRSA result. Microarray-based characterisation (*S. aureus* Genotyping Kit 2.0, Abbott, Alere Technologies, Jena, Germany) detected *mecA* and assigned the isolate (Graz_511421–19) to clonal complex CC1-MRSA-IV. Although the antibiotic treatment was adapted, the patient died shortly after because of tumour progression.

## Isolates

Ten CC1-MRSA-IV isolates originated from the Sfanta Parascheva Hospital, Iasi, in north-eastern Romania [4]. Four isolates originated from the Dresden University Hospital, Saxony, Germany. Nineteen isolates from Regensburg University Medical Centre in Bavaria, Germany and five isolates from other Bavarian hospitals were also included. All included isolates had been collected and preliminarily analysed as part of earlier collaborations. Four fully sequenced reference strains were used as controls (Table 1). Additional controls comprised four isolates from Dresden that belonged to local epidemic strains or to another CC1-MRSA strain (isolate Dresden-220663).

**Table 1:** MRSA strains and isolates investigated in the present study and their detection using commercially available *orfX*/SCC*mec* junction site assays (n = 47)

Isolate	Strain affiliation (according to microarray)	SCC*mec* element	Origin	BD MAX results	GeneXpert results
Graz_511421-19 (index case)	CC1-MRSA-IV (PVL-neg, *aphA3/sat*-pos)	SCC*mec* IVa with insertion	Austria, 2019	Negative (G)	Negative (2 X BC; G)
Dresden-94757	CC1-MRSA-IV (PVL-neg, *aphA3/sat*-pos)	SCC*mec* IVa with insertion	Saxony, 2010	Negative (D, G)	N/A
Dresden-94758	CC1-MRSA-IV (PVL-neg, *aphA3/sat*-pos)	SCC*mec* IVa with insertion	Saxony, 2014	Negative (D, G)	N/A
Dresden-94759	CC1-MRSA-IV (PVL-neg, *aphA3/sat*-pos)	SCC*mec* IVa with insertion	Saxony, 2009	Negative (D, G)	N/A
Dresden-94760	CC1-MRSA-IV (PVL-neg, *aphA3/sat*-pos)	SCC*mec* IVa with insertion	Saxony, 2010	Negative (D, G)	N/A
Iasi-95033	CC1-MRSA-IV (PVL-neg, *aphA3/sat*-pos)	SCC*mec* IVa with insertion	Romania, 2009	Negative (D, G)	N/A
Iasi-95034	CC1-MRSA-IV (PVL-neg, *aphA3/sat*-pos)	SCC*mec* IVa with insertion	Romania, 2009	Negative (D)/ambiguous (G)^a^	N/A
Iasi-95035	CC1-MRSA-IV (PVL-neg, *aphA3/sat*-pos)	SCC*mec* IVa with insertion	Romania, 2009	Negative (D)/ambiguous (G)^a^	N/A
Iasi-95037	CC1-MRSA-IV (PVL-neg, *aphA3/sat*-pos)	SCC*mec* IVa with insertion	Romania, 2009	Negative (D, G)	N/A
Iasi-95038	CC1-MRSA-IV (PVL-neg, *aphA3/sat*-pos)	SCC*mec* IVa with insertion	Romania, 2009	Negative (D, G)	N/A
Iasi-95039	CC1-MRSA-IV (PVL-neg, *aphA3/sat*-pos)	SCC*mec* IVa with insertion	Romania, 2009	Negative (D)/ambiguous (G)^a^	N/A
Iasi-95040	CC1-MRSA-IV (PVL-neg, *aphA3/sat*-pos)	SCC*mec* IVa with insertion	Romania, 2009	Negative (D, G)	N/A
Iasi-95041	CC1-MRSA-IV (PVL-neg, *aphA3/sat*-pos)	SCC*mec* IVa with insertion	Romania, 2009	Negative (D, G)	N/A
Iasi-174752	CC1-MRSA-IV (PVL-neg, *aphA3/sat*-pos)	SCC*mec* IVa with insertion	Romania, 2010	Negative (D, G)	N/A
Iasi-176047	CC1-MRSA-IV (PVL-neg, *aphA3/sat*-pos)	SCC*mec* IVa with insertion	Romania, 2009	Negative (D, G)	N/A
Bavaria-0643	CC1-MRSA-IV (PVL-neg, *aphA3/sat*-pos)	SCC*mec* IVa with insertion	Bavaria, 2018	N/A	Positive (SSTI; R)
Bavaria-0824	CC1-MRSA-IV (PVL-neg, *aphA3/sat*-pos)	SCC*mec* IVa with insertion	Bavaria, 2015	N/A	Positive (SSTI; R)
Bavaria-1185	CC1-MRSA-IV (PVL-neg, *aphA3/sat*-pos)	SCC*mec* IVa with insertion	Bavaria, 2018	N/A	Positive (SSTI; R)
Bavaria-1274	CC1-MRSA-IV (PVL-neg, *aphA3/sat*-pos)	SCC*mec* IVa with insertion	Bavaria, 2014	N/A	Positive (SSTI; R)
Bavaria-1537	CC1-MRSA-IV (PVL-neg, *aphA3/sat*-pos)	SCC*mec* IVa with insertion	Bavaria, 2013	N/A	Positive (SSTI; R)
Bavaria-1780	CC1-MRSA-IV (PVL-neg, *aphA3/sat*-pos)	SCC*mec* IVa with insertion	Bavaria, 2013	N/A	Positive (SSTI; R)
Bavaria-1962	CC1-MRSA-IV (PVL-neg, *aphA3/sat*-pos)	SCC*mec* IVa with insertion	Bavaria	N/A	Positive (SSTI; R)
Bavaria-2102	CC1-MRSA-IV (PVL-neg, *aphA3/sat*-pos)	SCC*mec* IVa with insertion	Bavaria, 2019	N/A	Positive (SSTI; R)
Bavaria-2220	CC1-MRSA-IV (PVL-neg, *aphA3/sat*-pos)	SCC*mec* IVa with insertion	Bavaria	N/A	Positive (SSTI; R)
Bavaria-2312	CC1-MRSA-IV (PVL-neg, *aphA3/sat*-pos)	SCC*mec* IVa with insertion	Bavaria	N/A	Positive (SSTI; R)
Bavaria-2360	CC1-MRSA-IV (PVL-neg, *aphA3/sat*-pos)	SCC*mec* IVa with insertion	Bavaria	N/A	Positive (SSTI; R)
Bavaria-2391	CC1-MRSA-IV (PVL-neg, *aphA3/sat*-pos)	SCC*mec* IVa with insertion	Bavaria, 2018	N/A	Positive (SSTI; R)
Bavaria-2483	CC1-MRSA-IV (PVL-neg, *aphA3/sat*-pos)	SCC*mec* IVa with insertion	Bavaria, 2019	N/A	Positive (SSTI; R)
Bavaria-2535	CC1-MRSA-IV (PVL-neg, *aphA3/sat*-pos)	SCC*mec* IVa with insertion	Bavaria, 2019	N/A	Positive (SSTI; R)
Bavaria-2584	CC1-MRSA-IV (PVL-neg, *aphA3/sat*-pos)	SCC*mec* IVa with insertion	Bavaria, 2019	N/A	Positive (SSTI; R)
Bavaria-2585	CC1-MRSA-IV (PVL-neg, *aphA3/sat*-pos)	SCC*mec* IVa with insertion	Bavaria, 2019	N/A	Positive (SSTI; R)
Bavaria-2588	CC1-MRSA-IV (PVL-neg, *aphA3/sat*-pos)	SCC*mec* IVa with insertion	Bavaria, 2019	N/A	Positive (SSTI; R)
Bavaria-2596	CC1-MRSA-IV (PVL-neg, *aphA3/sat*-pos)	SCCmec IVa with insertion	Bavaria, 2019	N/A	Positive (SSTI; R)
Bavaria-2618	CC1-MRSA-IV (PVL-neg, *aphA3/sat*-pos)	SCC*mec* IVa with insertion	Bavaria, 2012	N/A	Positive (SSTI; R)
Bavaria-3012	CC1-MRSA-IV (PVL-neg, *aphA3/sat*-pos)	SCC*mec* IVa with insertion	Bavaria, 2011	N/A	Positive (SSTI; R)
Bavaria-3254	CC1-MRSA-IV (PVL-neg, *aphA3/sat*-pos)	SCC*mec* IVa with insertion	Bavaria, 2010	N/A	Positive (SSTI; R)
Bavaria-3702	CC1-MRSA-IV (PVL-neg, *aphA3/sat*-pos)	SCC*mec* IVa with insertion	Bavaria, 2019	N/A	Positive (SSTI; R)
Bavaria-3741	CC1-MRSA-IV (PVL-neg, *aphA3/sat*-pos)	SCC*mec* IVa with insertion	Bavaria, 2019	N/A	Positive (SSTI; R)
Bavaria-3784	CC1-MRSA-IV (PVL-neg, *aphA3/sat*-pos)	SCC*mec* IVa with insertion	Bavaria, 2019	N/A	Positive (SSTI; R)
Dresden-220663	CC1-MRSA-IV, (PVL-neg, *aphA3/sat*-neg)	SCC*mec* IVa (as in reference strain MW2)	Saxony, 2007	Positive (D)	N/A
Dresden-124288	CC22-MRSA-IV (Barnim/UK EMRSA-15)	SCC*mec* IVh/j	Saxony	Positive (D)	N/A
Dresden-124289	CC22-MRSA-IV (Barnim/UK EMRSA-15)	SCC*mec* IVh/j	Saxony	Positive (D)	N/A
Dresden-124281	CC45-MRSA-IV (Berlin EMRSA)	SCC*mec* IVa	Saxony	Positive (D)	N/A
MU50	CC5-MRSA-II (New York/Japan clone)	SCC*mec* II	Japan (sequenced reference strain)	Positive (D)	N/A
MW2	CC1-MRSA-IV (PVL-pos US400)	SCC*mec* IVa	United States (sequenced reference strain)	Positive (D)	N/A
N315	CC5-MRSA-II (New York/Japan clone)	SCC*mec* II	Japan (sequenced reference strain)	Positive (D)	N/A
US300-FPR3757	CC8-MRSA-[Iva-pos ACME1] (PVL-pos ), US300	SCC [*mec* IVa + ACME1 + Cu]	US (GenBank CP000255.1)	Positive (D)	N/A

BC: GeneXpert MRSA/SA BC; CC: clonal complex; MRSA: meticillin-resistant *Staphylococcus aureus*; EMRSA: epidemic strain of MRSA; N/A: not available; PVL: Panton–Valentine leukocidin; SCCmec: staphylococcal cassette chromosome mec; SSTI: GeneXpert MRSA/SA SSTI.

D, G, and R indicate that assays were performed in Dresden, Graz and Regensburg, respectively.

^a^ Ambiguous: weak signal observed at cycle threshold Ct > 35.

## Commercial MRSA assays

Test results are provided in Table 1. The index isolate tested negative using the BD MAX Staph SR assay (Lot 9303156). Isolates from Iasi and Dresden and controls were tested twice, in Graz and Dresden, using this assay (Graz, Lot 9303156; Dresden, Lot K55928980720210312). It failed to identify these 15 isolates although controls handled in parallel were correctly identified.

Testing of the index isolate with GeneXpert MRSA/SA BC (Lots 1000148707 and 1000179462) yielded negative results, too. Further investigations on this assay were not possible because that laboratory became involved in diagnostics for the coronavirus (Covid-19) pandemic.

All Bavarian isolates were tested using Cepheid GeneXpert MRSA/SA SSTI (Lot 1000180532) that gave correct, positive results.

## Genotyping by microarray and sequencing

All isolates were genotyped using the *S. aureus* Genotyping Kit 2.0, a microarray covering 333 different target sequences corresponding to ca 170 different genes. Target genes, assay protocols and sequences of probes and primers have been published previously [5]. Isolates were assigned to clonal complexes, strains and SCC*mec* types based on microarray data as described [5].

All isolates underwent whole-genome sequencing. DNA was extracted as for array experiments. Its quality was assessed as previously described [6]. The Nextera DNA Flex Library Preparation Kit (Illumina, Eindhoven, the Netherlands) was used and libraries underwent paired-end sequencing using the 500-cycle MiSeq Reagent Kit v2 (Illumina). Libraries were scaled to exhibit at least 50-fold coverage. Sequencing run quality was assured following cluster density and Q30 assessment. Raw sequence reads were trimmed using fastp 0.19.11 [7] and assembled using SPAdes v3.9.1 [8]. Contigs under 1,000 bp were removed.

The sequence of the SCC*mec* element one representative isolate, Iasi-95037, was deposited in GenBank (accession number: MT380478).

## Description of the strain and its SCC*mec* element

Microarray profiling and genome sequencing showed that the index isolate belonged to a CC1-MRSA-IV clone previously described as ‘European CC1-MRSA-IV’ that may have emerged in south-eastern Europe [1,4,9]. A putative, meticillin-susceptible ancestor is common in Romania where this MRSA clone frequently observed already several years ago [1,4]. A high prevalence or outbreaks have been reported from Ireland [1], Italy [10] and Germany (North Rhine-Westphalia and Bavaria) [1,11]. In Regensburg, retrospective microarray-based typing of 3,067 isolates revealed that the occurrence of the European CC1-MRSA-IV clone increased from < 1% of typed MRSA between 2010 and 2013 to 9.4% in 2019. In Dresden, this strain has only sporadically been observed, accounting for seven in 1,758 isolates genotyped since 2000 ([1,12] and data not shown). Microarray genotyping data indicated that this clone was also recovered from horses and wild birds in Austria [13,14] and from livestock in Italy [15].

Isolates of this clone typically exhibit sequence type (ST)1 (1–1-1–1-1–1-1) or ST4110 (1–1-1–1-1–1-558) and *spa* types t127 (07–23–21–16–34–33–13), t386 (07–23–13) or t13790 (07–23–21–16–34–33–34–34–33–34). Isolates usually carry *ermC* (erythromycin/clindamycin resistance), *tetK* (tetracycline resistance), *aphA*3 (kana-/neomycin resistance), *aadE* (streptomycin resistance) and *sat* (streptothricin resistance). Some isolates harbour *aacA-aphD* (gentamicin resistance). Isolates from Ireland frequently exhibit resistance to mupirocin, chlorhexidine and quaternary ammonium compounds because of plasmid-borne *iles2/mupR* and *qacA* [1]. Fusidic acid resistance has not yet been detected in this clone, in contrast to other CC1-MRSA of from the Middle East or the southern hemisphere.

The clone is PVL-negative and lacks the *splE* protease gene. It only rarely carries enterotoxin genes *sek/seq* in addition to *seh* that is ubiquitously present in CC1. Its relationship to other CC1-MRSA clones has been discussed previously in detail [1].

This CC1-MRSA clone has an SCC*mec* IVa element which is essentially identical in all isolates and in a previous Irish sequence (GenBank RBVO00000000.1) [1]. In contrast to MW2 (BA000033.2), it harbours an insertion of ca 5,350 nt, adjacent to *orfX* (Table 2, Figure). The insertion affects the *orfX/*SCC*mec* junction that is targeted by molecular tests for the detection of MRSA. It starts with a SCC terminal sequence alternate to *dcs* (‘SCCterm 15’) and encodes six hypothetical proteins (E7MHX1, *ydiL*2, C5QAP8, A8YYX4, *npd* and H4AYD7; RBVO000005.1: 280,690–286,024). This insertion replaces *dcs*/Q9XB68*-dcs* and removes most (212 of 240 nt) of a gene encoding hypothetical protein Q7A213.

**Table 2:** Genes in the variant SCC*mec* IVa element of the European CC1-MRSA-IV strain

Gene ID	Definition of gene product and comments (see also annotation of MH188467.1)	Orientation	Locus tag in MW2 (BA000033.2)	Nucleotide positions in GenBank RBVO	Nucleotide positions in GenBank MT380478 (Iasi-95037)
*orfX*	23S rRNA methyltransferase	Forward	MW0024	RBVO01000005.1; nt 280,209–280,689	N/A
sRNA6	Antisense RNA associated with orfX	Reverse	N/A	RBVO01000005.1; nt 280,389–280,673	N/A
DR_SCC	Direct repeat of SCC, to 19 nt of the 3' end of the coding sequence of orfX	N/A	N/A	RBVO01000005.1; nt 280,670–280,689	nt 1–19
sccterm15	SCC-terminal sequence adjacent to orfX, and alternate to dcs, see Discussion	N/A	Not present	RBVO01000005.1; nt 280,689–280,912	nt 20–242
E7MHX1	Transcription regulator	Forward	Not present	RBVO01000005.1; nt 280,912–281,239	nt 243–569
*ydiL2*	Hypothetical protein/putative membrane peptidase, associated with SCC elements	Forward	Not present	RBVO01000005.1; nt 281,275–282,109	nt 606–1,439
C5QAP8-M299	Hypothetical protein	Forward	Not present	RBVO01000005.1; nt 282,794–283,568	nt 2,125–2,898
A8YYX4	Hypothetical protein	Reverse	Not present	RBVO01000005.1; nt 283,805–284,144	nt 3,136–3,474
*npd-*SCC	Enoyl-[acyl-carrier-protein] reductase-like protein	Reverse	Not present	RBVO01000005.1; nt 284,329–285,400	nt 3,660–4,730
H4AYD7-trunc	Transcriptional regulator, LysR family	Truncated	Not present	RBVO01000005.1; nt 285,412–286,024	nt 4,743–5,354
Q7A213-trunc	Putative protein; it comprises the inverted repeat of IS431. In MW2 it is not truncated and comprises 240 nt	Truncated	MW0026	RBVO01000005.1; nt 286,024–286,052	nt 5,355–5,382
IR_IS431	Inverted repeat of IS431	Truncated	N/A	RBVO01000005.1; nt 286,024–286,040	nt 5,355–5,370
tnpIS431	Transposase for IS431	Reverse	MW0027	RBVO01000005.1; nt 286,083–end of contig (nt 286,184) (partial)	nt 5,414–6,088
Teg143	Trans-encoded RNA associated with tnpIS431	Forward	N/A	RBVO01000003.1; nt 203–237	nt 6,119–6,152
IR_IS431	Inverted repeat of IS431	Truncated	N/A	RBVO01000003.1; nt 213–229	nt 6,129–6,144
*mvaS-*SCC	Truncated HMG-CoA synthase	Forward	MW0028	RBVO01000003.1; nt 245–598	nt 6,161–6,513
Q5HJW6	Hypothetical protein	Forward	N/A	RBVO01000003.1; nt 695–1,046	nt 6,611–6,841
*dru*	SCC direct repeat units	Truncated	N/A	RBVO01000003.1; nt 835–1,273	nt 6,751–7,148
*ugpQ*	Glycerophosphoryl diester phosphodiesterase-like protein	Forward	MW0029	RBVO01000003.1; nt 1,474–2,218	nt 7,350–8,093
*ydeM*	Acyl dehydratase MaoC	Forward	MW0030	RBVO01000003.1; nt 2,314–2,743	nt 8,190–8,618
*mecA*	Encodes penicillin binding protein 2 prime, defining MRSA	Reverse	MW0031	RBVO01000003.1; nt 2,812–4,795	nt 8,664–10,670
*mecR1-*trunc	Meticillin resistance operon repressor 1, signal transducer protein, truncated in SCCmec IV	Truncated	MW0032	RBVO01000003.1; nt 4,894–5,862	nt 10,770–10,816
*hsdR2-*IS1272	Type I site-specific deoxyribonuclease restriction subunit	Truncated	MW0033	RBVO01000003.1; nt 5,869–6,103	nt 11,745–11,978
tnpIS1272	Transposase	Reverse	MW0034	RBVO01000003.1; nt 6,103–7,627	nt 11,979–13,502
Q9KX75	Hypothetical protein	Reverse	MW0035	RBVO01000003.1; nt 7,762–8,269	nt 13,638–14,144
Q7A207	Hypothetical protein	Reverse	MW0036	RBVO01000003.1; nt 8,283–8,595	nt 14,159–14,470
Q7A206-trunc	Hypothetical protein, truncated	Truncated	N/A	RBVO01000003.1; nt 8,596–8,683	nt 14,472–14,558
Q7A206	Hypothetical protein	Reverse	MW0037	RBVO01000003.1; nt 8,681–9,032	nt 14,557–14,907
UTR_*ccrB-2*	Highly conserved 3'-untranslated region of *ccrB*	N/A	N/A	RBVO01000003.1; nt 9,032–9,553	nt 14,908–15,428
*ccrB-2*	Cassette chromosome recombinase B2	Reverse	MW0038	RBVO01000003.1; nt 9,553–11,182	nt 15,429–17,057
*ccrA-2*	Cassette chromosome recombinase A2	Reverse	MW0039	RBVO01000003.1; nt 11,203–12,553	nt 17,079–18,428
*cch-2*	Hypothetical protein/cassette chromosome helicase	Reverse	MW0040	RBVO01000003.1; nt 12,786–14,574	nt 18,662–20,449
DUF1413	Hypothetical protein, associated with cch	Reverse	MW0041	RBVO01000003.1; nt 14,573–14,864	nt 20,449–20,739
Q2FKL7	Putative membrane protein	Forward	MW0042	RBVO01000003.1; nt 15,002–16,052	nt 20,878–21,927
Q8VUV8	Putative transcriptional regulator	Forward	MW0043	RBVO01000003.1; nt 16,504–17,995	nt 22,380–23,870
*cstB-*SCC2	Includes a putative beta-lactamase; marker for SCCm*ec* IVa	Truncated	MW0045	RBVO01000003.1; nt 18,367–19,685	nt 24,243–25,560
Q2FKL3	HNH endonuclease family protein	Forward	MW0046	RBVO01000003.1; nt 19,875–20,247	nt 25,751–26,122
Q8VUW0	Putative membrane protein	Forward	MW0047	RBVO01000003.1; nt 20,375–20,996	nt 26,251–26,871
DR_SCC	Direct repeat of SCC	Truncated	N/A	RBVO01000003.1; nt 21,300–21,319	nt 27,176–27,194

MRSA: meticillin-resistant *Staphylococcus aureus*; N/A: not available; nt: nucleotide position; SCC: staphylococcal cassette chromosome.

**Figure:**
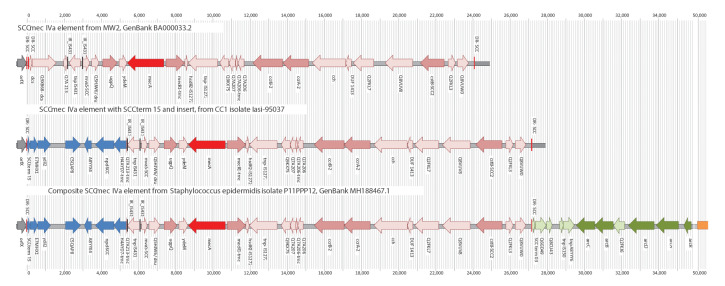
SCC*mec* elements in the CC1 reference sequence MW2, the European CC1-MRSA-IV isolate Iasi-95037and the *Staphylococcus epidermidis* isolate P11PPP12

GenBank accession numbers: MW2: BA000033.23; Iasi-95037: MT380478; P11PPP12: MH188467.1.

SCCterm 15 is present in at least three other MRSA strains. In CC152-MRSA-XIII, it is close to *orfX* (MG674089, CP024998), possibly affecting MRSA PCRs. In two other strains, it is situated within complex SCC*mec* elements [16]. In a Danish CC8-MRSA strain (HM030720.1), the same insert as in the European CC1-MRSA is localised between an ACME-II and an SCC*mec* IVa element [17,18]. A Saudi Arabian CC22-MRSA strain (HF569105.1) harbours SCCterm 15, E7MHX1, *ydiL*2, IR_IS431 and tnpIS431, localised between a copper resistance element and a composite ACME-II/ SCC*mec* IVh/j element [19].

An identical 5,350 nt cluster is present in *Staphylococcus epidermidis* P11PPP12 (MH188467.1). Beyond that, the entire SCC*mec* IV element in P11PPP12 is identical to the one in the CC1-MRSA strain. Significantly, the site of recombination cutting short Q7A213 is conserved in both strains (position 5,818/5,819 in the Supplement). Thus, it is likely that the *entire* SCC*mec* IVa cassette including the insert was transferred between ancestors of the two strains, i.e. across the species. However, P11PPP12 also harbours an ACME-II/heavy metal resistance element downstream of SCC*mec*, which is absent from the European CC1-MRSA-IV. Therefore, it must have been lost during or after transfer of the SCC*mec* element, or it was acquired later by *S. epidermidis*.

## Discussion

The study demonstrates that a CC1-MRSA-IV epidemic strain in Europe can yield false-negative results with common MRSA assays (GenXpert MRSA/SA BC, BD MAX Staph SR). Interestingly, GeneXpert MRSA/SA SSTI yielded correct results, indicating that the different tests utilise different primers. The absence of *dcs* and coverage of SCCterm 15 appear to be the reason for the discrepancy.

False-negative results of PCRs targeting the *orfX*/SCC*mec* junction site are concerning. Molecular assays are used to predict MRSA in positive blood cultures and to change therapy accordingly. The use of molecular assays is beneficial for a vast majority of patients because a result is available quickly. However, these assays can only detect target sequences that were available and considered at the time the primers were designed, and false-negative results have the potential to harm the patient by delaying effective therapy. Conventional antibiotic susceptibility tests are slower but are not constrained by the choice of primers or by the presence of unknown genotypes.

Molecular assays are also used to guide infection control. False-negative tests may result in lapses facilitating further MRSA transmission. Another, less obvious consequence might be a shift in the clonal structure of MRSA populations. When molecular assays exert a selective pressure favouring a false-negative strain, PCR-positive strains might get ‘penalised’ with subsequent interventions, hindering proliferation and transmission. This could lead to an increasing prevalence of the false-negative strain and to more failures in therapy and infection control.

It is crucial to monitor the emergence of new SCC*mec* junction sites in *S. aureus* and in coagulase-negative staphylococci, as mobile SCC*mec* elements can readily be transmitted between different strains and species, as was the case in the strain described here. Such unknown genotypes represent a problem for established molecular assays. As illustrated here, updating existing tests and platforms to evolving genotypes of the target organisms is important for individual and public health.

The containment of the CC1 strain must rely on conventional susceptibility tests, culture-based screening using selective growth media or updated molecular tests. We propose screening of medical or nursing staff recruited from epidemic regions, not only in hospitals but also in other care facilities, as well as patients with travel histories to these regions.

## Supplementary Data

1773000 Supplement
